# Water quality and resource management in the dairy industry

**DOI:** 10.1007/s11356-017-0608-8

**Published:** 2017-11-03

**Authors:** Joanna Boguniewicz-Zablocka, Iwona Klosok-Bazan, Vincenzo Naddeo

**Affiliations:** 1 Department of Thermal Engineering and Industrial Facilities, Faculty of Mechanical Engineering, Opole University of Technology, Opole, Poland; 2 Sanitary Environmental Engineering Division (SEED), Department of Civil Engineering, University of Salerno, Fisciano, Italy

**Keywords:** Dairy industry, Water consumption, Water quality, Resources management

## Abstract

Food industry is one of the most important and fastest growing sectors of economy in Poland. This sector is also characterized by high demand for the resources, particularly for water. Polish food industrial plants consumed 793 hm^3^ of water in 2014. Dairy branch had a combined 35% share of the above consumption. As shown by the data obtained from the Polish Central Statistical Office, the majority of dairy plants use its own source of water, so this branch is also important water producer in Poland. Water used for dairy industry should meet the requirements of at least drinking water quality, so the factories need to treat the water. This paper analyses the correlations between selected technical process, equipment profiles and water quality, and consumption in two types of dairy factories (DF). The first one DF-1 processes approx. 50,000 L of milk, and the second, DF-2 processes approx. 330,000 L of milk per day. The water taken from the wells needs to be pre-treated because of iron and manganese concentration and due to specific requirements in various industrial processes. As a result of this work, we have managed to propose technological solutions in the context of water consumption rationalization. The proposed solutions aim at improving water and wastewater management by reducing the amount of consumed water by industry.

## Introduction

Water is used in various areas of business and industry; therefore, it is subject to the regulations that control the market (Becker 2016). With regard to the industry, the food sector has one of the highest water consumption and is one of the biggest producers of effluent per unit of production. In addition, the food sector generates a large volume of sludge during biological wastewater treatment (Anielak 2008; BriãoI and Granhen Tavares 2007). Meanwhile, the availability of freshwater resources adequate in both quantity and quality is vital to food security and production (Kirby et al. 2003). The food industry, also in Poland, is one of the most important and fastest growing sectors of economy (Wojdalski et al. 2013). Looking at the Polish food industry in terms of water resources, it has consumed more than 793 hm^3^ of water resources in 2014. At the same time, in 2014, Polish industrial sector generated more than 7876 hm^3^ of sewage, out of which 734.5 hm^3^ falls in the dairy industry, as analyzed further in this article (Central Statistical Office 2015).

Poland is one of the major producers of milk in the EU, having an 8.3% share in the global production. Consumers emphasize that Polish milk is characterized by high quality and nutritional value. Among the EU countries, Poland is the fourth producer of milk, followed by Germany, France, and the Great Britain. In the world ranking, Poland ranks twelfth position (Olszewska 2015). The dairy industry due to the nature of the raw material and the processing technology affects primarily the quality of water, and less the air and soil (Salou et al. 2017).

The dairy industry consumes great amounts of water and generates large quantities of wastewater (Kirby et al. 2003, Andrade et al. 2014). The most dangerous is the technological wastewater formed during washing equipment and facilities. It contains the remains of milk or other milk products as well as cleaning products. It is characterized by great diversity of pollutant concentrations over time. It depends largely on the type of production in the enterprise and the production cycle, being in the most part a batch processes (Bednarski 1997; Pham et al. 2011). Under these circumstances, water resource management agendas are in need of a holistic approach to environmental management (Hermanowicz 2008). Whereas all the pro-environment actions, including those in the field of water management, should follow the rules from the BREF reference documents (BAT Reference Note 2008), in particular the following: the use of low-waste technology; the use of less hazardous substances; the furthering of recovery and recycling of substances generated and used in the process and of waste, where appropriate; technological advances and changes in scientific knowledge and its understanding; the consumption and nature of raw materials (including water) used in the process and their energy efficiency; and the need to prevent or reduce to a minimum the overall impact of the emissions on the environment and the risks to it. Problems of reducing water consumption, increasing the effectiveness of wastewater treatment, and implementation of adequate hygiene standards have been discussed by many authors. According to published research results, most dairy plants consume from 1 to 10 m^3^ of water per every cubic meter of milk production. The highest rates of water consumption are presented by Bosworth et al. (2000), who gathered the data representing Danish dairy enterprises. Additionally, the data used consisted of that presented by Vourch et al. (2008) from the French market; data presented in the BAT for the Nordic dairy industry (2002, 2005) in the Nordic countries Sweden, Finland and Norway; and the data presented by Wojdalski et al. (1987, 2013) from Polish dairy enterprises. The lowest rates of water consumption from dairy plants were observed in Australia, as presented by Prasad et al. (2004). The comparison of average rates of water consumption per each liter of processed milk for seven selected countries is to be found in Table 1.

**Table 1:** Indicators of dairy plant water consumption in selected countries

Country	Indicators L water/L milk
Denmark	2.21–9.44
Poland	3.48–9.77
France	0.20–10.00
Australia	1.05–2.21
Sweden	0.96–4.00
Finland	1.20–4.60
Norway	2.05–6.30

Source: Author’s own elaboration based on presented literature review

Presumably, the most important element of proper water management in the food industry is to ensure proper water quality for individual processes. The most important ways of water use in the dairy industry consist the technological processes, cleaning systems, cooling systems, steam generators, fire protection systems, and water used for social purposes.

Depending on the quality of water and the technical requirements of use, this water has to be further adjusted to suit different needs (Fleiflec et al. 2014), such as removal of color, softening or the addition of chlorine to minimize the count of potential spoilage microorganisms, or the use of UV radiation, e.g., to disinfect stored water directly before use as an ingredient (Casani et al. 2005). Water, alike food, can be a potential vehicle for the direct transmission of disease agents and continues to cause significant outbreaks of disease in both developed and developing countries. For example, drinking water was identified as the source of a significant and fatal outbreak of *Escherichia coli* in Canada (Kondro 2000). Water is, therefore, also capable of introducing contamination into food if appropriate care is not taken. In 2006, a chocolate company located in the UK was affected by contamination of chocolate by Salmonella, reputedly from a leaking wastewater pipe in the production area (Podolak et al. 2010).

Health risk can also be caused by a variety of chemical substances present in water and food products, which is different than risk caused by bacteriological contamination. There is a small chance that a single substance could cause acute poisoning (except for emergency situations). However, given the fact that a number of chemicals in concentrations normally found in water can cause negative health effects with long-term exposure, toxic substances having the ability to accumulate in the body, as well as substances with carcinogenic properties, are also subject to strict rigor and are eliminated from water intended for human and animal consumption. This is due to sanitary standards on water quality, among which the most important legislation in the European Union being the Directive of the Parliament and the EU Council on the quality of water intended for human consumption, specifying the standard chemical, physical, and biological properties which must be met by water supplied and used in food plant production (EU Regulation 1998).

Water cannot pose even a potential hazard to human health. The Directive defines both of the aforementioned parameters of permissible concentrations of substances harmful to the human body (toxic, mutagenic, and carcinogenic compounds and substances), and parameters such as color, turbidity, total number of bacteria, total organic carbon, taste, and smell. Although the parameters have no direct impact on consumer health, the aim is to determine the effectiveness and quality of water treatment process, which is referred to as auxiliary indicator parameter. It should be noted that the standards for a number of indicators applicable under the Directive are more restrictive than the WHO recommendations (Mulik et al. 2011). However, the proper management of water resources should not be applied only to water used for food processing. The dairy industry is characterized also by a significant water consumption for example for heat exchange. The adequate preparation of cooling water and the use of innovative biocides may improve the efficiency of water resources (Podgórni et al. 2015). In addition, water management is linked to many policy areas, and their integration is essential for the sustainable use of water (Aymerich et al. 2015). Particularly relevant in this context are segments of industry where the good state of aquatic ecosystems is relevant to maintain an adequate state of people’s health.

The aim of this study was to analyze two dairy enterprises particularly taking into account sustainable management of water resources. The paper is presented with case studies. It was considered that the case studies are based on the grounded theory of methodology and are deepening the knowledge that cannot be obtained by other means in order to explain phenomena that have not yet been explained and refer to specific companies.

## Materials and methods

The scope of the research includes a description of each of the analyzed companies; the analysis of their legal requirements in the field of water management; the data collected on the functioning of the plant: production, volume, and quality of water consumed (including water consumption in various technological processes); the flow and load of the sewage; existing practices and procedures for water management; and analysis of the obtained results. The procedure for water quality sampling and the appliances used for water quality parameter measurements were determined according to recommendations of Polish State Sanitary Inspection. The spectrophotometer manufactured by HACH Co. model DR 2000 (Hach, Loveland, CO, USA) was applied for water quality analysis of ammonium, nitrate, nitrite, iron, and manganese turbidity and color. The hydrogen ion concentration (pH) was measured with a pH meter CP—411 (ELMETRON, Gliwice, Poland), and the electrical conductivity was measured using a conductivity meter—CC—411 (ELMETRON, Gliwice, Poland). Hardness level was determined by titration following accredited testing procedure. The concentration of total count of microorganisms was conducted as per PN-EN ISO 6222:2004. The measurement of smell has been conducted via a simplified method based on the norm PN-EN 1622. Some data was taken from direct interview with the employees of the company. Furthermore, the correlations between selected technical process and production factors, equipment profiles, water quality, and consumption are also presented. The collected data was compared with the data available in literature. It should be noted that the analyzed companies had diverse production profiles and management procedures.

The first factory (DF-1) processes approx. 50,000 L of milk per day. The DF-1 is characterized by own drill well and additional water supply system connection with the municipal network, so that in case of accident or increased production, the water could be charged from municipal water supply. The presented dairy cooperative uses water for technological purposes, washing plant and equipment, steam generation and heating, as well as partly for social purposes. Average daily water demand is *Q*_*d*_ = 127 m^3^/day, the average hourly demand is *Q*_*h*_ = 5 m^3^/h, whereas the maximum is *Q*_maxd_ = 140 m^3^/day. Because of increased color and iron concentration, the water is subject to pre-treatment process. Following the treatment, it is transported into a clean water tank with the capacity of *V* = 48 m^3^, and through the use of pressure tank, the water is directed to the factory water supply system. The wastewater produced in the process of filter backwashing is discharged into industrial treatment plant.

DF-1 is in the possession of a permit for the uptake of groundwater. The individual amounts of pollutants in the wastewater discharged into the sanitary sewage system determine other water permit disposed by the facility. At the production facility, there are three types of sewer network, namely the sanitary sewage system, storm water network, and the technological wastewater network. The storm water system is used to drain rainwater from the site. Dairy effluent is pre-treated in industrial wastewater treatment plant. Dairy processing discharge wastewater characterized by high organic load due to the presence of milk components. Wastewater are characterized by high changes in temperature or pH. Other parameters of importance for dairy effluent are high COD, TSS, N, and P. As indicated also by Borbon et al. (2014), high BOD_5_ and suspended solids are the main pollutants that arise from leaks, spills, and removal of adhering materials during cleaning and sanitizing of equipment, cleaning and sanitizing solution with water washing and cooling water.

The second factory (DF-2) processes approx. 330,000 L of milk per day. At DF-2, the water supply system is based on its own underground water intake. Water intakes are located approximately 0.75–1.00 km from the plant. Noteworthy is the fact that a slight amount of water produced by the industry can be sold to the third parties. Water intake, as in the case of a DF-1, includes a defined zone of primary and intermediate protection. DF-2 owns a water permit for groundwater intake originating from their own sources serving for production and social purposes, as issued by the head of district. The consumption amounts are as follows: the daily average *Q*_avd_ = 2.16 m^3^/day, the hourly average *Q*_avh_ = 90 m^3^/h, and the hourly maximum *Q*_maxh_ = 180 m^3^/h. Raw water from wells is transported under pressure by pumps for drinking water treatment plant, where it is subject to processes of removing iron and manganese and chlorination. Then, it is pumped into two underground reservoirs with a capacity of 500 m^3^ each and subsequently distributed throughout the whole plant as treated drinking water. Water used for the supply and charging of boilers requires additional preparation. This includes processes such as filtration, softening, and reverse osmosis. The DF-2 factory makes use of CIP systems, which limits the consumption of raw water by reusing the water from the last rinse of equipment to the first rinse cycle. DF-2 also owns water permit for underground water uptake and wastewater permit to discharge wastewater. If whey from the cheese-making process is not used as a by-product and discharged along with other wastewaters, the organic load of the resulting effluent is further increased (Janczukowicz et al. 2008).

The dairy processing plants manufacture various dairy products where the primary ingredient is raw milk. In this paper, the presented case studies covered DF-1—milk production and DF-2—cheese production line. The processes taking place at the milk plant include milk receipt and filtration of raw milk; separation of complete or part of the milk fat due to standardization of market milk; pasteurization and homogenization, if required; followed by packaging and storage, including refrigerated storage. In the cheese production, line milk is separated into skim milk and cream, then pasteurized, followed by specific processes depending on the desired product. The product is packaged and stored before being distributed.

**Fig. 1:**
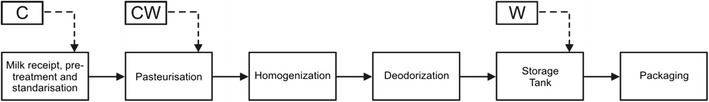
Flow diagram for processes occurring at a fluid milk production plant where C cleaning and sanitization solutions, CW cooling water, W water wash

**Fig. 2:**
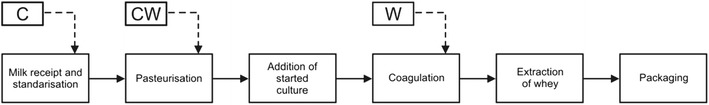
Flow diagram for processes occurring at a cheese plant where C cleaning and sanitization solutions, CW cooling water, W water wash

Selected processes employed for manufacturing of various products are indicated in Fig. 1 for fluid milk, and Fig. 2 for cheese production, respectively. The finished products are then packaged, cased, and sent to storage for subsequent shipment. The diagrams indicate places where high water consumption occurs for technological processes and for washing of machines and devices.

## Results and discussion

### Water quality

The basic element of proper water management in the food industry is to ensure adequate water quality. This is a prerequisite for prevention of incidents arising from security threats. Risk prevention constitutes of detection and identification (recognition). Risk control in both factories is carried out by specially developed programs to monitor the quality of raw water and treated water. Such programs provide appropriate steps throughout the whole chain: supply, production, and distribution of water. The most important, however, insignificant from the point of view of the analysis of sanitary water security threats is a parameter that requires constant monitoring, namely—iron. Iron as a component of water-bearing rocks occurs in waters of almost 80% underground sources. Harmless from the point of view of health, it causes significant trouble in production processes, particularly affecting negatively the quality of dairy products. The most important parameters of the quality of raw water in individual plants with reference to the current standards are presented in Table 2.

**Table 2:** The most important water quality parameters in DF-1 and DF-2 wells, with reference to the current national and European standards

Parameter	Unit	Raw water in DF1	Raw water in DF2	European Legislation Directive 98/83/EC	Polish Legislation Regulation 2015/1989/PL
Color	–	12 (mgPt/L)	24	Acceptable to consumers and no abnormal change	Acceptable to consumers and no abnormal change
Turbidity	NTU	14	20	Acceptable to consumers and no abnormal change	1
Odor	–	Acceptable and no abnormal change	Hydrogen sulphide - minimal perceptible	Acceptable to consumers and no abnormal change	Acceptable to consumers and no abnormal change
Hydrogen ion concentration	pH	7.2	6.9	6.5–9.5	6.5–9.5
Conductivity	μS cm^−1^ at 20 °C	567	624	2500	2500
Ammonium	mg/L	0.015	0.31	0.50	0.5
Nitrate	mg/L	0.23	0.30	50	50
Nitrite	mg/L	0.009	0.007	0.50	0.50
Hardness	mg /L	320	287	–	60–500 (CaCO_3_)
Iron	μg/L	1070	1810	200	200
Manganese	μg/L	170	137	50	50
*Escherichia coli*	number/100 ml	0	0	0	0
Enterococci	number/100 ml	0	0	0	0
Clostridium perfringens(including spores)	number/100 ml	0	0	0	0

Since at both plants the problem of excessive concentration of iron in raw water has been recognized, both analyzed plants are equipped with iron removal system. Iron removal systems operate on the basis of the same unit processes, namely, aeration, which aims to oxidize ferrous iron, as well as rapid filtration, in an attempt to stop the precipitated iron compounds. In order to ensure microbiological safety after the iron removal process, at both plants, the water is being disinfected with the use of chlorine. Analysis of archival data and interviews with employees at the plant lead to the conclusion that the operating treatment system ensures the required security level, both in terms of quantity and quality of water used in the manufacturing process. As previously mentioned, the process water, which may have contact with food, must meet strict quality requirements, which prevents it from being reused.

Nonetheless, the water used for washing machines and halls, especially when used for the first washing, is not the subject of such restrictive preconditions. This case was adopted at DF-2 where the CIP system has been implemented. After the completion of the technological process, clearing machines, and equipment from the product and its detachment from supply tanks, the rinsing is conducted in a closed circuit. The process of cleaning the line begins with pre-wash, which removes the remaining product from washed surfaces. Cleaning agent containing many contaminants is removed from the system. Thereafter, the preliminary heavily contaminated water is transported directly to the treatment plant. The initial washing is followed by proper wash with the use of detergents. The solution remaining from last washing is passed to the pre-wash tank at the washing station. The process ends with rinsing with clean water. Considering that such water may have contact with food production, it must meet the requirements for drinking water.

Other requirements are set for the water used for steam production. In the dairy industry, the technological steam is used not only as a heat carrier, but also, and perhaps primarily as a disinfecting agent in the process of sterilization and pasteurization. Some processes require direct contact of steam with the product (e.g., a direct sterilization process for producing the UHT milk). If in the production process the steam being used has direct contact with food products, the parameters of water used for steam production should correspond to the parameters of drinking water, including the boiler water used for steam boilers. Such water must meet the requirements defined by the manufacturers of boilers, which usually relate to the hardness of water. The requirements depend on the design of a boiler and increase accordingly with its operating pressure.

At both study sites, there are water softener stations which effectively remove hardness to the levels below 0.01 mval/l, supporting the operation of boilers. In addition, water in the boiler system is adjusted through the use of chemicals that do not have toxic, carcinogenic, mutagenic, or harmful properties and are thermostable under normal operating conditions of the boiler. These substances are approved by the National Institute of Health for having contact with food. Both basic ingredients of these formulas and complementarity-stabilizing additives are a category of chemicals listed in food additives. This approach of both companies creates the comfort of safety, both from the point of view of the construction of the boiler and steam quality, and thus the quality of dairy products.

Another problem regarding water management at the company is to ensure the supply of water to maintain greenness in the summer, and the provision of sufficient quantities of water in case of fire. In these cases, the requirements for water quality are not as restrictive, since this part of water management depends on the quantity of water available. At both plants during the summer months, the maintenance of green areas was carried with raw water and additionally with treated wastewater which quality allowed for such use (Zahuta 2015). Regarding the fire objectives, the capacity of the reservoirs of clean water does not guarantee complete safety in the event of a large fire. Therefore, companies should consider the possibility of using treated wastewater as a potential reservoir for fire protection. The quality of treated wastewater allows such a solution; however, it should be noted that the water should contain disinfectant components, so that when sprayed during a water rescue, it does not pose a bacteriological danger for persons conducting work at the company.

### Water consumption indicators

Dairy processors are aggressively challenged to conserve water necessitating the need for not only reducing water consumption but also to employ measures for recovery and recycling of process water without compromising on the hygienic quality and safety of the products. In Poland, there are norms of water consumption specified by the Minister of Infrastructure Regulation of 14 January 2002 on determining the average water consumption standards. Specific standards of the average water consumption for dairy products are presented in Table 3.

**Table 3:** Average water consumption standards in processing plants in dairy industry based on Regulation (2002)

Type of dairy industry	Unit of reference (ur)	Average water consumption indicators, m^3^/ur
Dairy industry		
Butter production	1000 dm^3^ mleka	3.0
Cheese production	1000 dm^3^ mleka	4.0
Milk receipt and storage	1000 dm^3^ mleka	0.3

**Fig. 3:**
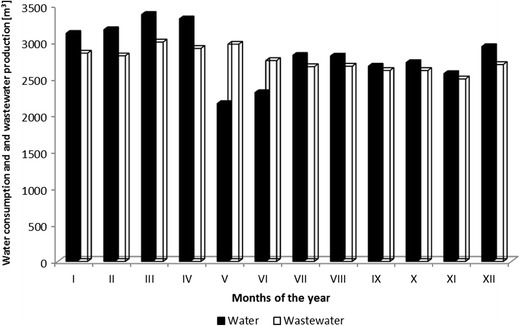
Changes in monthly water consumptions and wastewater production during the year

In the literature, Flemmer (2012), Perry (2011) and Steinhoff-Wrześniewska et al. (2013), Strzelczyk et al. (2010), there is diversified information on the use of water, which typically oscillates between 1 ÷ 10 l of water per liter of processed milk. However, the actual water consumption varies depending on the progress and modernization of production facilities and introduction of new technologies. In the production of milk and dairy products, one can observe a certain itineracy. Just as diverse is the demand market for dairy products during a year, accordingly varied is the demand for water and the volume of wastewater discharged from these processes. This is presented in Fig. 3, which shows the changes in monthly water consumption and wastewater production during a year. As can be seen, the volume of wastewater is equivalent to about 90% of water production, while during the summer, this figure is somehow lower, because a small amount of treated wastewater is used for irrigation of green areas.

Respective processes have been analyzed at the plants in order to evaluate water consumptions. Water consumption indicators broken down by technological processes in both analyzed companies are presented in Tables 4 and 5.

**Table 4:** Water consumption indicators for DF-1 in 2014

Process	Unit	Value
Milk pre-treatment	m^3^ for m^3^ proceeded milk	0.4
Milk processing and standardization	m^3^ for m^3^ proceeded milk	0.5
Pasteurization	m^3^ for m^3^ proceeded milk	0.9
Cleaning operations	m^3^ for m^3^ proceeded milk	1.2
Employees sanitary facilities	m3 for m3 proceeded milk	0.8
∑	m3 for m3 proceeded milk	3.8

**Table 5:** Water consumption indicators for DF-2 in 2014

Process	Unit	Value
Milk pre-treatment	m3 for m3 proceeded milk	0.3
Cheese production processes	m3 for m3 proceeded milk	0.8
Pasteurization	m3 for m3 proceeded milk	0.9
Cleaning operations	m3 for m3 proceeded milk	1.8
Employees sanitary facilities	m3 for m3 proceeded milk	0.8
∑	m3 for m3 proceeded milk	4.6

The diversity of indicators at individual factories was discovered to be resulting from the diversity in the type of production. When comparing the data with the available literature data as presented in the introduction, at Table 1, it can be noted that the situation at the analyzed companies is not drastic, but there is a potential to reduce water consumption from primary sources. The capability for the introduction of innovative technologies can be applied for such operations as automated rinsing of milk receiving tank trucks and cleaning of the tankers at the dairy plant; automated dairy food processing operations processing milk; automated circulation cleaning; and the use of liquid detergents and chemical sanitizing agents on a controlled basis.

The data presented indicate a high potential of this industry to use systems reuse water. According to literature, data recovery of water used can save up to 20–40% of the total costs associated with the production of water (Milani et al. 2011; Kasztelan 2012).

This is an essential fact because Poland is in the process of major legislative changes and the introduction of significant increase in fees for the use of water from primary sources. Therefore, any change will translate into tangible economic savings and improve enterprise competitiveness.

Undoubtedly, water consumption is also influenced by the nature of dairy production. Most water is required at plants that produce milk powder and cheese, a little less is consumed at plants producing drinking milk. In order to compare the levels of water consumption for both of analyzed plants, the average water consumption indicators have been calculated: the average for years 2011–2014 and for 2015 only, after the introduction of water management industry changes. The data are presented in Table 6.

**Table 6:** Water consumption indicators: average for 2011–2014 and for 2015

Plants	Amount of processed milk during the year (average 2011–2015) [L]	Water consumption indicator (average 2011–2014) [L for L proceeded milk]	Water consumption indicator 2015 [L for L proceeded milk]
DF1	50,000	3.5 +/− 0.3	3.2
DF2	330,000	4.7 +/− 0.9	4.0

When taking into account data of the year by year period, we may observe reduction in water consumption per liter of product. These improvements are attributed to developments in process control and cleaning practices. During the research period on presented case studies has been implemented the system of simple changes and lower water consumption in the washing process. Additionally, some percent of wastewater has been used for green area irrigation, and all leaks have been monitored. At DF-1, 10% decrease in water consumption indicator has been achieved. At DF-2, almost 15% presumably because DF-2 uses CIP and only a slight modification to CIP has resulted in high reduction of fresh water use. Significant impact of controlling and optimizing cleaning parameters on water consumption was described also by Wojdalski et al. (2013). Practical solutions for reducing water consumption in dairy plants indicate that the water scarcity footprint at the plants was not only related to total freshwater consumption and production, but also closely related to the scarcity of water resources in the watershed basin or area (Bai et al. 2017).

The above data allows us to draw very optimistic forecasts and conclusions, namely that the existing measures aimed to reduce water consumption from primary sources do bring the expected results.

## Conclusions

The problems of environment protection in the industrial sectors are becoming more and more relevant, with strict legal requirements that imply considerable investments. This encourages researchers to look for new systemic solutions and methodologies to improve efficiency of water management. Industrial plants are specific in terms of quantity and quality of treated water, applied technologies and technical solutions, and specific operational regime. Thus, the decision about selecting the most appropriate type of technology is very individual and should be accurate for selected industrial plant.

Management strategies for improving water productivity of dairy production have to start at the source of the water. Since water treatment technology significantly influences the total consumption of water at the industrial plants, we should properly select the source of water and unit processes, so that water consumption for technological purposes is as low as possible.

Further technologies used in production require large quantities of water, used either for washing machine, cooling system, or product processing. The possible ways these operations can be modified or employee practices changed to reduce water use are identified and discussed. The role of management in processing water and waste control is an important factor for rational water management in the industry. In the literature, we could find a water consumption rate even of 1.3–2.5 m^3^ water/m^3^ of milk intake (Wojdalski et al. 2013); however, in order to achieve such low consumption, the advanced technology and very good housekeeping and awareness among both employees and management are necessary.

## References

[CR1] Andrade LH, Mendes FDS, Espindola JC, Amaral MCS (2014). Nanofiltration as tertiary treatment for the reuse of dairy wastewater treated by membrane bioreactor. Sep Purif Technol.

[CR2] Anielak A (2008). Dairy industry and its water-sewage economics. Agro Przemysł.

[CR3] Aymerich I, Rieger L, Sobhani R, Rosso D, Corominas L (2015). The difference between energy consumption and energy cost: modelling energy tariff structures for water resource recovery facilities. Water Res.

[CR4] Bai X, Ren X, Khanna NZ, Zhou N, Hu M (2017) Comprehensive water footprint assessment of the dairy industry chain based on ISO 14046: a case study in China. Resources, Conservation and Recycling. Online 3 August 2017. 10.1016/j.resconrec.2017.07.021

[CR5] BAT Guidance Note (2002) Best available techniques for the Nordic dairy industry. http://eldri.ust.is/media/skyrslur2002/BAT_mjolkuridn_2001-586.pdf

[CR6] BAT Guidance Note (2005). Best available techniques. Guidelines for the dairy industry. The National Fund for Environmental Protection and Water Management.

[CR7] BAT Guidance Note (2008). Best available techniques for the dairy processing Sector.

[CR8] Becker RA (2016). Water use and conservation in manufacturing: evidence from U.S. microdata. Water Resour Manag.

[CR9] Bednarski W (1997). Dairying. Selected Issues.

[CR10] Borbon B, Oropeza-Guzman MT, Brillas E, Sires I (2014). Sequential electrochemical treatment of dairy wastewater using aluminum and DSA-type anodes. Environ Sci Pollut Res.

[CR11] Bosworth M, Hummelsmose B, Christiansen K (2000). Cleaner production assessment in dairy processing.

[CR12] BriãoI V, Granhen Tavares C (2007) Effluent generation by the dairy industry: preventive attitudes and opportunities. Braz J Chem Eng. 10.1590/S0104-66322007000400003

[CR13] Casani S, Rouhanyb M, Knøchela S (2005). A discussion paper on challenges and limitations to water reuse and hygiene in the food industry. Water Res.

[CR14] Central Statistical Office (2015) Environment 2014. Warszawa pp157 (in Polish). http://stat.gov.pl/obszary-tematyczne/srodowisko-energia/srodowisko/ochrona-srodowiska-2015,1,44.html

[CR15] Fleiflec A, Saavedra O, Yoshimura C, Elzeir M, Tawfik A (2014). Optimization of integrated water quality management for agricultural efficiency and environmental conservation. Environ Sci Pollut Res.

[CR16] Flemmer C (2012). Environmental input–output analysis of the New Zealand dairy industry. Int J Sustain Dev.

[CR17] Hermanowicz SW (2008). Sustainability in water resources management: changes in meaning and perception. Sustain Sci.

[CR18] Janczukowicz W, Zielinski M, Debowski M (2008). Biodegradability evaluation of dairy effluents originated in selected sections of dairy production. Bioresour Technol.

[CR19] Kasztelan A (2012). The impact of the food industry on the environment in Poland. Przem Spożywczy.

[CR20] Kirby RM, Bartram J, Carr R (2003). Water in food production and processing: quality and quantity concerns. Food Control.

[CR21] Kondro W (2000). E coli outbreak deaths spark judicial inquiry in Canada. Lancet.

[CR22] Milani F, Nutter D, Thoma G (2011). Environmental impacts of dairy processing and products: A review. J Dairy Sci.

[CR23] Mulik B, Stankiewicz A, Wichrowska B (2011) Problems with the implementation of the Drinking Water Directive, ENDWARE conference materials available on http://www.pis.gov.pl/userfiles/file/Depar-tament%20BW/Artyku%C5%82y/11_2011Problemy%20z%20wdra%C5%BCaniem%20DWD.pdf (in Polish)

[CR24] Olszewska M (2015). Milk production in Poland compared to the world and European Union countries. Wiadomości Zootechniczne.

[CR25] Perry C (2011). Accounting for water use: terminology and implications for saving water and increasing production. Agric Water Manag.

[CR26] Pham MPT, Castle JW, Rodgers JH (2011). Application of water quality guidelines and water quantity calculations to decisions for beneficial use of treated water. Appl Water Sci.

[CR27] Podgórni E, Boguniewicz-Zablocka J, Klosok-Bazan I (2015). The impact of nano-silver doses on microorganism-deactivation effectiveness in water circulating in a cooling tower cycle. Pol J Environ Stud.

[CR28] Podolak R, Enache E, Stone W, Black DG, Elliott PH (2010) Sources and risk factors for contamination, survival, persistence, and heat resistance of Salmonella in low-moisture foods. J Food Prot 73:1919–1936 10.4315/0362-028x-73.10.1919 21067682

[CR29] Prasad P, Pagan R, Kauter M, Price N (2004). Eco-efficiency for the dairy processing industry. Environmental Management Centre.

[CR30] Regulation EU (1998) Council Directive 98/83/EC of 3 November 1998 on the quality of water intended for human consumption. http://eur-lex.europa.eu/legal-content/EN/TXT/?uri=CELEX%3A31998L0083

[CR31] Regulation RP (2002) Regulation of infrastructure minister on determining the average water consumption standards (Official Journal Nro.8, Item 70)

[CR32] Salou T, Le Mouel CH, Van der Werf H (2017). Environmental impacts of dairy system intensification: the functional unit matters. J Clean Prod.

[CR33] Steinhoff-Wrześniewska A, Rajmund A, Godzwon J (2013). Water consumption in selected branches of food industry. Inż Ekol.

[CR34] Strzelczyk M, Steinhoff-Wrześniewska A, Rajmund A (2010). Indicators of water consumption and the quantity of wastewater formed in selected branches of food industry. Pol J Chem Technol.

[CR35] Vourch M, Balannec B, Chaufer B, Dorange G (2008). Treatment of dairy industry wastewater by reverse osmosis for water reuse. Desalination.

[CR36] Wojdalski J, Michnikowska-Plucińska T, Zdun K, Malejko B (1987). Dairy industry and its water-sewage economics. Gosp Wodna.

[CR37] Wojdalski J, Dróżdż B, Piechocki J, Gaworski M, Zander Z, Marjanowski J (2013). Determinants of water consumption in the dairy industry. Pol J Chem Technol.

[CR38] Zahuta K (2015) Water and waste water management in the plant. Master’s degree thesis. Opole University of Technology (in Polish)

